# Circulating Biomarkers and Recurrence of Persistent Atrial Fibrillation After Electrical Cardioversion

**DOI:** 10.1177/11772719251361306

**Published:** 2025-09-11

**Authors:** Elizabeth Lyster Andersen, Magnar Gangås Solberg, Steve Enger, Sophia Onarheim, Mona Olufsen, Trygve Berge, Sissel Åkra, Maiken Kojen Kleveland, Ingrid Elisabeth Christophersen, Sara Reinvik Ulimoen, Ingebjørg Seljeflot, Arnljot Tveit

**Affiliations:** 1 Department of Medical Research, Bærum Hospital, Vestre Viken Hospital Trust, Gjettum, Norway; 2 Institute of Clinical Medicine, Faculty of Medicine, University of Oslo, Norway; 3 Center for Clinical Heart Research, Department of Cardiology, Oslo University Hospital Ullevål, Norway; 4 Department of Medical Genetics, Oslo University Hospital, Norway

**Keywords:** biomarkers, atrial fibrillation, electrical cardioversion, rhythm outcomes, AF recurrence, rhythm control

## Abstract

**Background:**

Rhythm control therapy is recommended for individuals with symptomatic atrial fibrillation (AF) to reduce symptoms and improve quality of life. Electrical cardioversion (ECV) is used to restore sinus rhythm (SR), but AF recurrence is common.

**Objective:**

We aimed to investigate if a selection of circulating biomarkers can predict rhythm outcomes in individuals with persistent AF treated with ECV.

**Design:**

This was an observational cohort study.

**Methods:**

We included 200 individuals aged ⩾ 18 years referred for ECV of AF from November 2017 to March 2022. We obtained blood samples 0 to 6 weeks before ECV. Plasminogen activator inhibitor type 1 (PAI-1) activity, soluble suppression of tumorigenicity 2 (sST2), galectin-3 (GAL-3), interleukin-6 (IL-6), matrix metalloproteinase-9 (MMP-9), tissue inhibitor of metalloproteinase-1 (TIMP-1), growth differentiation factor-15, (GDF-15), transforming growth factor-β-1 (TGF-β1), and fibroblast growth factor-23 (FGF-23) were analyzed by ELISA methods. The participants recorded thumb ECGs twice daily for 28 days after the ECV to detect AF recurrence.

**Results:**

A total of 188 individuals were eligible for the analyses. Twenty-four participants converted spontaneously to SR before ECV. Among the cardioverted, 74 maintained SR, whereas 90 experienced AF recurrence before hospital discharge (n = 15) or during the follow-up period of 28 days (n = 75). TIMP-1 was significantly higher in those with AF recurrence than in those who maintained SR, but overlapping distributions suggest limited predictive ability. PAI-1 activity, sST2, GAL-3, IL-6, MMP-9, GDF-15, TGF-β1, and FGF-23 did not differ among the participants who had ECV.

**Conclusion:**

TIMP-1 was higher in participants with recurrence of AF after ECV, but its predictive ability was limited. None of the other biomarkers were associated with AF recurrence. We do not recommend using these biomarkers for candidate selection for ECV of persistent AF.

## Plain Language Summary

Atrial fibrillation (AF) is the most common heart arrhythmia, often causing symptoms and leading to reduced quality of life. Electrical cardioversion is a method used to restore normal heart rhythm in patients with persistent AF; however, about half of the patients experience AF recurrence within a few weeks after the electrical cardioversion. In this observational study, we examined whether a set of blood biomarkers measured before electrical cardioversion could predict rhythm outcomes four weeks after the procedure. We found that TIMP-1 levels were higher in participants with AF recurrence than in those who maintained normal heart rhythm. However, the TIMP-1 levels overlapped between the groups, and its predictive ability was limited. We recommend not using the studied biomarkers to guide which patients should undergo electrical cardioversion for persistent AF.

## Central Illustration


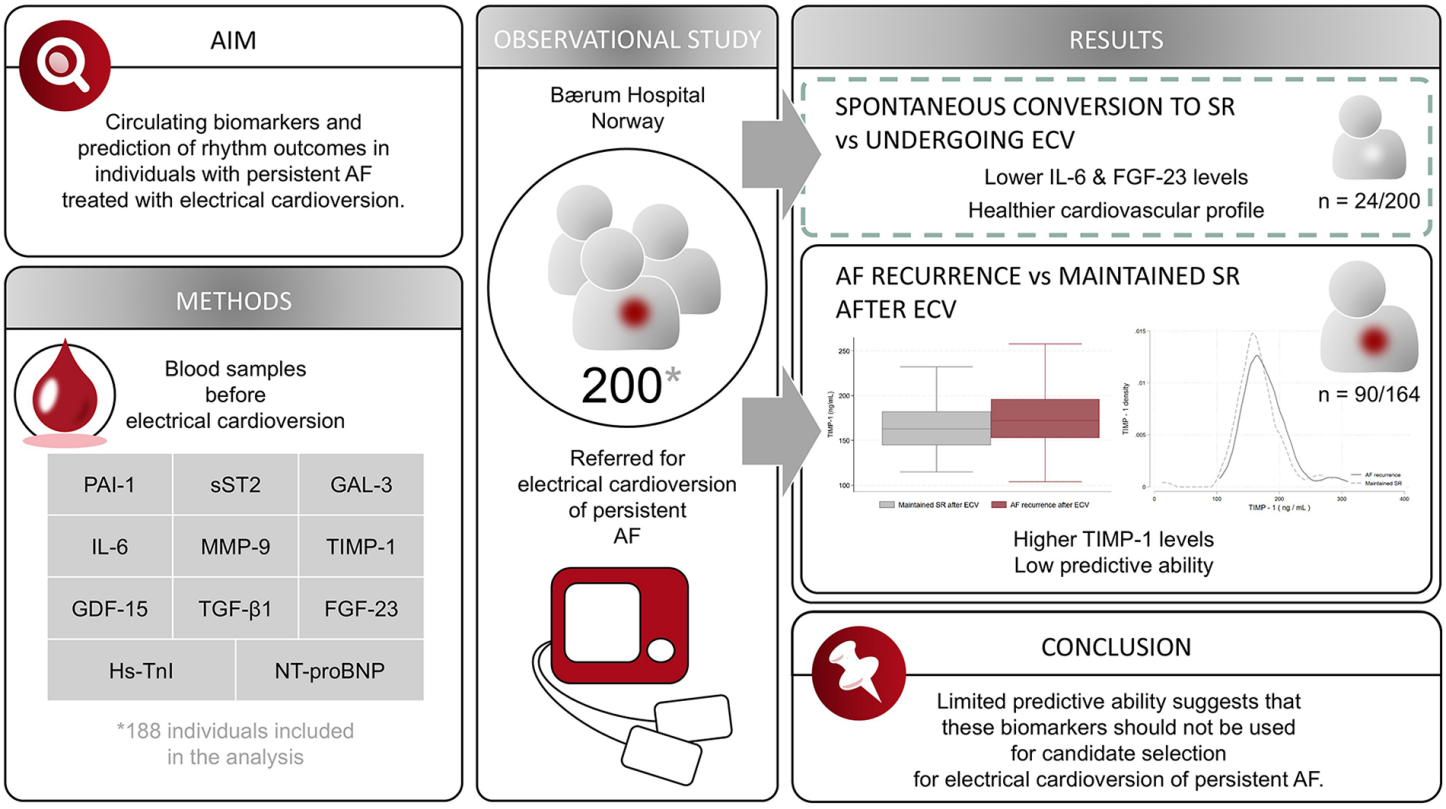


## Introduction

Atrial fibrillation (AF) is the most common sustained arrhythmia worldwide.^1^ AF lasting ⩾ 7 days is classified as persistent and may need intervention to be terminated. The European Society of Cardiology (ESC) AF guidelines recommend rhythm control therapy in patients with AF-related symptoms to reduce symptoms and improve quality of life.^2^ Electrical cardioversion (ECV) is an effective method for terminating persistent AF. More than 90% of patients achieve sinus rhythm (SR) immediately after the procedure, but 32% to 62% of the successfully cardioverted patients experience a recurrence of AF within a few weeks after the ECV.^3-5^

The guidelines highlight an evidence gap in personalized AF risk prediction.^2^ It has been suggested that atrial fibrosis plays an essential role in atrial remodeling in AF pathophysiology.^6-8^ Cardiac fibrosis is characterized by increased deposition of extracellular matrix proteins in the myocardial tissue and may be triggered by myocardial injury, stress, and inflammation.^9^

High-sensitivity troponin I (hs-TnI) is released during myocardial injury,^10^ and N-terminal pro-B-type natriuretic peptide (NT-proBNP) increases in reaction to pressure or volume overload.^11^ Both are used in clinical practice and may be helpful tools for improving the treatment of AF. Plasminogen activator inhibitor type 1 (PAI-1) activity levels are involved in coagulation, fibrinolysis, and inflammation and are increased in fibrotic tissues.^12^ Studies have shown increased PAI-1 activity^13^ and upregulated PAI-1 protein expression^14^ in individuals with AF compared to healthy controls. Suppression of tumorigenicity 2 (ST2), a member of the IL-1 receptor family, exists in a soluble (sST2) and a transmembrane (ST2L) isoform, both shown to be upregulated in myocardial stress and injury.^15^ Galectin-3 (GAL-3) is a beta-galactoside binding protein expressed by infiltrating myofibroblasts in cardiomyocytes and other cell types and is involved in inflammation and fibrosis.^16^ A meta-analysis showed increased GAL-3 levels in individuals with AF compared to healthy controls and an association with AF progression.^17^ Interleukin-6 (IL-6) is a cytokine with pro-inflammatory and anti-inflammatory effects.^18^ Previous studies have reported increased IL-6 levels in individuals with AF compared to those in SR^7,19^ and an association with AF progression.^19^

Matrix Metalloproteinase-9 (MMP-9) and its inhibitor Tissue Inhibitor of Metalloproteinase-1 (TIMP-1) are involved in the extracellular matrix formation.^20^ Alteration of MMPs and TIMPS are associated with cardiovascular diseases,^21^ and studies suggest a relation between AF burden and MMP-9/TIMP-1 levels.^19,22-24^ Growth Differentiation Factor-15 (GDF-15) is associated with LA overload, inflammation, and cellular homeostasis.^25^ Elevated GDF-15 is reported in individuals with cardiovascular diseases and is a marker associated with cardiovascular and non-cardiovascular death.^26-28^ Transforming Growth Factor-β-1 (TGF-β1) participates in tissue homeostasis.^29^ TGF-β1 is upregulated in individuals with AF^30^ and may be a potential predictor for AF recurrence after ECV. Fibroblast Growth Factor-23 (FGF-23) is secreted by osteocytes involved in phosphorus regulation, vitamin D metabolism, and bone mineralization.^31^ FGF-23 has been associated with cardiovascular diseases and AF.^32,33^

Several studies have reported associations between biomarkers and AF recurrence after ECV, but the findings are conflicting (Supplemental Table S1). We aimed to investigate whether a selection of biomarkers with potential roles in cardiac remodeling obtained before ECV predicted rhythm outcomes in individuals with persistent AF.

## Materials and Methods

### Design and Study Population

In an observational cohort study (PRE-ELECTRIC; predictors for recurrence of atrial fibrillation after electrical cardioversion), we enrolled 200 patients scheduled for ECV between November 2017 and March 2022 at Bærum Hospital, Norway. The inclusion criteria were being over 18 years of age and able to give consent. There were no exclusion criteria. The reporting of this study conforms to the STROBE statement^34^ (Supplemental File 1).

### Data Collection

**Figure 1.**
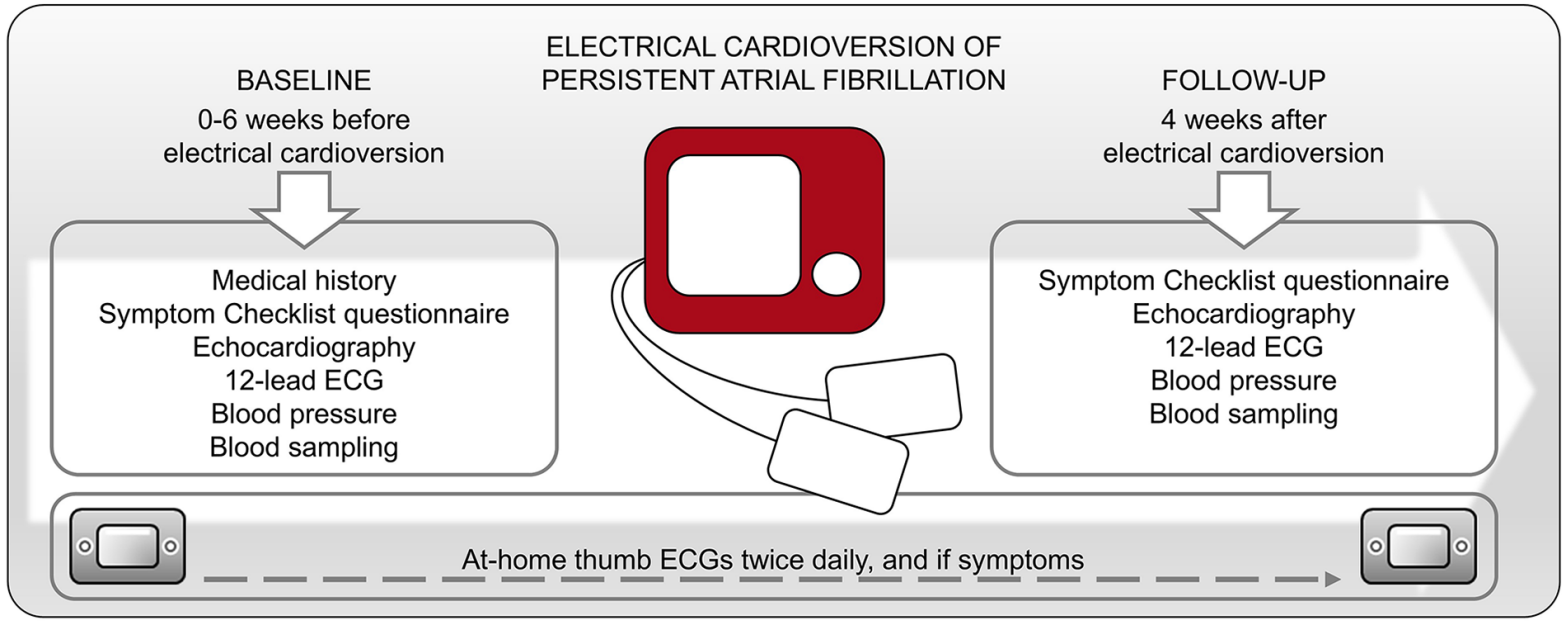
Data collection before and after ECV of persistent AF. The participants recorded thumb ECGs twice daily and if they experienced symptoms. Abbreviations: ECV, electrical cardioversion; ECG, electrocardiogram; AF atrial fibrillation.

We performed a physical examination, transthoracic echocardiography (TTE), 12-lead ECG, and blood sampling during the baseline visit prior to the scheduled ECV (Figure 1). We measured blood pressure after 10 minutes of rest in the supine position and took the average of 3 measurements. AF symptoms were classified according to the modified European Heart Rhythm Association (mEHRA) score, and the participants reported AF symptoms by answering the questionnaire “The Symptom Checklist: Frequency and Severity Scale” (SCL). The participants used a Zenicor© thumb ECG device to record ECGs routinely twice daily for 28 days following the ECV. They were instructed to record additional ECGs if they experienced any symptoms during this period. AF was classified as persistent if it was detected in ECGs for 7 consecutive days. TTE was performed using a Vivid E9 scanner (GE Healthcare, Horten, Norway), and images were stored digitally for offline analysis using custom software (EchoPac, GE Vingmed, Horten, Norway). Left ventricular (LV) dimension and septal wall thickness were measured according to the American Society of Echocardiography and the European Association of Cardiovascular Imaging.^35^ LV ejection fraction (LVEF) and left atrial volumes were calculated using 2D 4-chamber and 2-chamber views with the modified Simpson’s rule. Maximal left atrial volume (LAVImax) was measured at ventricular end-systole and indexed to body surface area.

### Laboratory Analysis

We collected fasting blood samples using the standard venipuncture procedure. Serum was prepared after coagulation and centrifugation at 2000*g* for 15 minutes at room temperature within 2 hours. EDTA and citrated plasma were kept on ice and centrifuged at 1920*g* for 20 minutes at 4°C within 30 minutes. Plasma and serum were pipetted into aliquots and frozen at −80°C in a biobank stored at Bærum Hospital. All samples were analyzed in batches to minimize variation. Routine blood samples, including hs-TnI and NT-proBNP, were analyzed at Bærum Hospital. Other biomarkers were analyzed at the Oslo Center for Clinical Heart Research Laboratory at Oslo University Hospital, Ullevål. All laboratory personnel performing biomarker analysis were blinded to the rhythm outcomes. For samples exceeding the maximum detection limit, the value was set to the highest detectable value. For samples below the minimum detection limit, the value was set to the lowest detectable value.

Hs-TnI and NT-proBNP were determined in serum samples using a Siemens Atellica immunoassay analyzer with commercially available reagent kits produced by Siemens Atellica. Standards of known concentrations were tested in duplicate, and quality controls were performed. Samples with concentrations lower than the measuring range were analyzed twice. The analytical coefficient of variation (CV) was 8% for hs-TnI and 9% for NT-proBNP.

Serum was used for the determinations of MMP-9, TIMP-1, GAL-3, GDF-15, and IL-6, all measured using ELISA kits from Bio-Techne, R&D Systems Europe (Abingdon, Oxon, UK; Human Quantikine MMP-9, Human Quantikine TIMP-1, Human Quantikine Galectin-3, Human Quantikine GDF-15, High sensitivity Quantikine Human IL-6) and ST2, measured by Presage ST2 Assay (Critical Diagnostics, San Diego, US). EDTA plasma was used to determine Human Quantikine TGF-β1 with a pre-activation step (Bio-Techne) and Human Intact FGF-23 (QUIDEL, San Diego, California, USA). PAI-1 activity was measured in citrated plasma by Spectrolyse PAI-1 (BioMedica Diagnostics Inc., Wentworth Road, Windsor, Canada). The CVs were 5.9% for MMP-9, 3.8% for TIMP-1, 3.9% for GAL-3, 4.3% for GDF-15, 6.1% for IL-6, 3.3% for sST2, 6.0% for TGF-β1, 9.5% for FGF-23, and 10.8% for PAI-1.

### Statistical Analysis

Normally distributed continuous data are presented as mean ± standard deviation (SD), whereas non-parametric continuous data are presented as median and interquartile range (IQR). Student’s *t*-tests were used to compare differences in normally distributed, continuous data. The Wilcoxon rank-sum test was used to compare differences in non-parametric data. Categorical data are presented as frequency and percentage, and differences were tested using the chi-square test. *P*-values < .05 were considered statistically significant. Biomarkers of special interest were each examined separately with multivariate logistic regression models adjusted for a set of confounders and visually presented in a coefficient plot. Missing data in the SCL questionnaires were replaced by person mean. The Spearman’s rho (rs) correlation coefficients were Bonferroni-adjusted to account for multiple comparisons, used for continuous, ratio, or ordinal variables of any distribution, and presented in a correlation matrix. We analyzed the time to AF recurrence and biomarker levels by quartiles using Kaplan-Meier plots and log-rank tests. Receiver Operating Characteristic (ROC) curves were used to assess the true positive rate against the false positive rate for variables of interest. A Kernel density plot was used to visualize the data distribution.

All statistical analyses were performed using STATA 17 (StataCorp).

## Results

**Figure 2.**
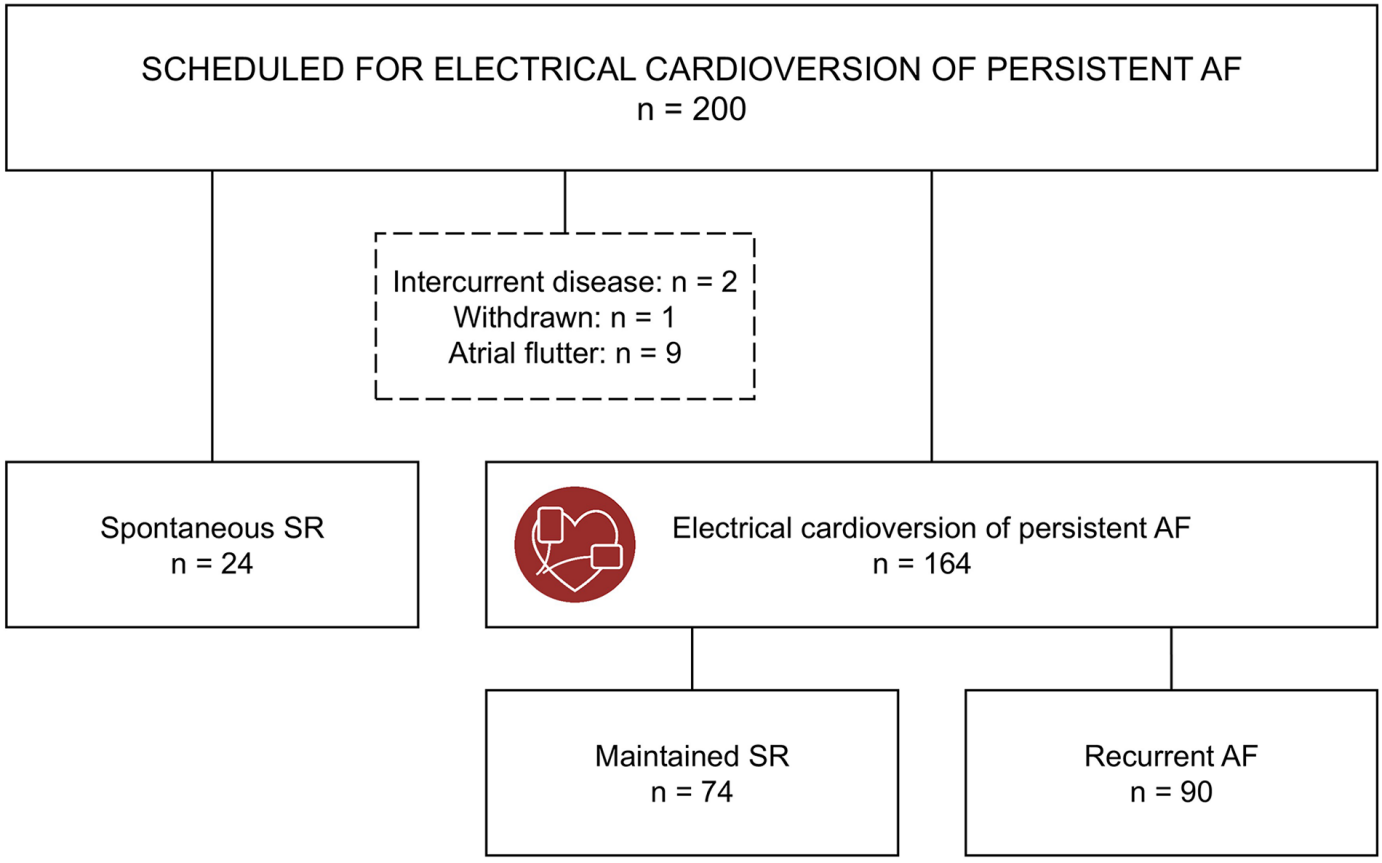
Study flow chart. Abbreviations: AF, atrial fibrillation; SR, sinus rhythm.

A total of 188 individuals were eligible for analysis (Figure 2). In the total population, the mean age was 66.0 ± 9.3 years, and 38 (20.2%) were women (Table 1). The mean systolic blood pressure was 126 ± 15 mmHg, and the body mass index (BMI) was 27.9 ± 4.5 kg/m^2^. Hypertension was the most common comorbidity (n = 88; 46.8%), followed by heart failure (n = 48; 25.5%) and ischemic heart disease (n = 19; 10.1%).

**Table 1.** Baseline Characteristics in Participants With Spontaneous Conversion to SR Versus Participants. Undergoing ECV.

	Total	Spontaneous SR	Undergoing ECV	*P*-value
Baseline Characteristics	n = 188	n = 24	n = 164	
Male sex	150 (79.8%)	19 (79.2%)	131 (79.9%)	.94
Age, years	66.0 (9.3)	64.0 (9.7)	66.3 (9.2)	.25
mEHRA score				.76
1	13 (6.9%)	3 (12.5%)	10 (6.1%)	
2a	24 (12.8%)	2 (8.3%)	22 (13.4%)	
2b	134 (71.3%)	17 (70.8%)	117 (71.3%)	
3	11 (5.9%)	1 (4.2%)	10 (6.1%)	
4	6 (3.2%)	1 (4.2%)	5 (3.0%)	
Symptom Check List, frequency	14.8 (9.1)	12.5 (6.8)	15.2 (9.3)	.18
Symptom Check List, severity	12.5 (7.7)	10.5 (6.3)	12.7 (7.9)	.18
Hypertension	88 (46.8%)	9 (37.5%)	79 (48.2%)	.33
Ischemic heart disease	19 (10.1%)	2 (8.3%)	17 (10.4%)	.76
Heart failure	48 (25.5%)	1 (4.2%)	47 (28.7%)	.01
Diabetes	13 (6.9%)	2 (8.3%)	11 (6.7%)	.77
TIA	10 (5.3%)	2 (8.3%)	8 (4.9%)	.48
Stroke	16 (8.5%)	2 (8.3%)	14 (8.5%)	.97
Systolic blood pressure (mmHg)	126 (15)	123 (11)	126 (15)	.36
Diastolic blood pressure (mmHg)	80 (12)	73 (7)	81 (12)	.001
Body Mass Index (kg/m^2^)	27.9 (4.5)	26.8 (3.7)	28.1 (4.6)	.20
Ventricular heart rate (BPM)	77 (17)	57 (11)	79 (16)	<.001
Antiarrhythmic drugs				
Flecainide	5 (2.7%)	0 (0.0%)	5 (3.0%)	.39
Betablocker	115 (61.2%)	14 (58.3%)	101 (61.6%)	.76
Amiodarone	29 (15.4%)	5 (20.8%)	24 (14.6%)	.43
Amiodarone^a^	35 (18.6%)	5 (20.8%)	30 (18.3%)	.77
Dronedarone	6 (3.2%)	0 (0.0%)	6 (3.7%)	.34
Non-dihydropyridine calcium channel blocker	4 (2.1%)	1 (4.2%)	3 (1.8%)	.46
Tobacco				.19
Never smoker	84 (44.7%)	14 (58.3%)	70 (42.7%)	
Current smoker	13 (6.9%)	0 (0.0%)	13 (7.9%)	
Previous smoker	91 (48.4%)	10 (41.7%)	81 (49.4%)	
Alcohol units per week	5.0 (2.0-8.0)	5.0 (1.0-10.0)	5.0 (2.0-8.0)	.98

Abbreviations: AF, atrial fibrillation; SR, sinus rhythm; ECV, electrical cardioversion; mEHRA, modified European Heart Rhythm Association symptom classification for AF. 1, None. 2a, Mild, normal activity not affected, not troubled. 2b, Moderate, normal activity not affected, but troubled. 3, Severe, normal activity affected, 4, Disabling, normal activity discontinued; TIA, transient ischemic attack; BPM, beats per minute.

Data are presented as mean (SD) or median (IQR) for continuous measures, and n (%) for categorical measures.

a Including patients with amiodarone startup after ECV.

All participants were diagnosed with persistent AF before being referred for elective ECV. Twenty-three out of 24 participants had spontaneously converted to SR by the baseline visit, with one more converting in the days before the planned ECV. Initially, we investigated whether the participants with spontaneous SR differed from those who required an ECV (Tables 1 and 2). Subsequently, we examined baseline characteristics and biomarkers by rhythm outcomes in the participants treated with ECV for persistent AF (Tables 3 and 4).

**Table 2.** Circulating and Echocardiographic Biomarkers at Baseline in Participants With Spontaneous Conversion to SR Versus Participants Undergoing ECV.

	Total	Spontaneous SR	Undergoing ECV	*P*-value
Biomarkers	n = 188	n = 24	n = 164	
Routine blood samples				
Hb (g/dL)	15.1 (14.3-16.0)	14.8 (13.9-15.9)	15.1 (14.3-16.0)	.28
CRP (mg/L)	4.0 (3.0-4.0)	4.0 (4.0-4.0)	4.0 (3.0-4.0)	.16
Sodium (mmol/L)	141 (139-142)	140 (138-141)	141 (139-142)	.08
Potassium (mmol/L)^a^	4.2 (3.9-4.4)	4.1 (3.8-4.2)	4.2 (3.9-4.4)	.07
Creatinine (µmol/L)	82 (74-90)	74 (66-84)	82 (75-92)	.01
eGFR (ml/min/1.73m^2^)	82 (72-90)	90 (78-90)	81 (70-90)	.03
Glucose (mmol/L)^a^	5.6 (5.2-6.2)	5.5 (5.2-6.0)	5.6 (5.2-6.2)	.43
HbA1c (mmol/mol)^a^	39 (36-42)	37 (35-40)	39 (36-42)	.11
Cholesterol total (mmol/L)	4.5 (3.8-5.3)	4.8 (4.2-5.5)	4.5 (3.8-5.2)	.16
Triglycerides (mmol/L)	1.1 (0.8-1.5)	1.1 (0.8-1.3)	1.1 (0.8-1.6)	.27
HDL (mmol/L)	1.3 (1.1-1.6)	1.6 (1.3-2.0)	1.3 (1.1-1.6)	.02
LDL (mmol/L)^a^	2.8 (2.1-3.6)	3.0 (2.4-3.7)	2.7 (2.1-3.6)	.30
Hs-TnI (ng/L)^a^	6.8 (4.4-10.6)	6.1 (4.2-13.9)	6.8 (4.5-10.1)	.53
NT-proBNP (ng/L)^a^	1042.7 (553.5-1838.4)	164.1 (124.2-338.6)	1137.8 (691.9-1940.8)	<.001
Potential novel biomarkers				
PAI-1 activity (IU/mL)^a^	9.9 (3.3-25.5)	8.7 (2.6-15.7)	10.4 (3.3-28.9)	.20
sST-2 (ng/mL)^a^	28.8 (23.8-37.8)	28.6 (23.0-32.7)	28.8 (24.0-37.9)	.44
GAL-3 (ng/mL)^a^	8.39 (6.81-10.66)	8.73 (7.39-10.96)	8.39 (6.70-10.64)	.36
IL-6 (pg/mL)^a^	2.55 (1.56-4.12)	1.68 (1.24-3.23)	2.57 (1.66-4.20)	.02
TIMP-1 (ng/mL)^a^	167 (150-189)	156 (141-183)	168 (151-192)	.25
MMP-9 (ng/mL)^a^	218 (170-295)	245 (210-326)	214 (166-293)	.06
GDF-15 (pg/mL)^a^	1022 (779-1375)	854 (745-1360)	1026 (796-1380)	.21
TGF-β1 (pg/mL)^b^	2488 (2142-2940)	2452 (2211-2753)	2488 (2128-2950)	.85
FGF-23 (pg/mL)^b^	60 (48-74)	52 (43-60)	61 (49-75)	.02
Echocardiographic biomarkers				
LAVImax, (ml/m^2^)^c^	48 (39-55)	39 (32-46)	49 (40-55)	<.001
LVEF (%)^a^	52 (48-55)	55 (52-57)	52 (45-54)	<.001
LVIDd (mm)^b^	52 (48-56)	52 (49-56)	52 (48-56)	.24
IVSd (mm)^b^	9 (8-10)	8 (8-10)	10 (9-11)	.01

Abbreviations: Hb, hemoglobin; CRP, C-reactive protein; Estimated GFR, estimated glomerular filtration rate; HbA1c, hemoglobin A1c (glycated hemoglobin); HDL, high-density lipoprotein cholesterol; LDL, low-density lipoprotein cholesterol; hs-TnI, high-sensitivity troponin I; NT-proBNP, N-terminal pro B-type natriuretic peptide; FT4, “free” thyroxine 4; TSH, Thyroid-stimulating hormone; PAI-1 activity, Plasminogen Activator Inhibitor type 1 activity; sST-2, soluble suppression of tumorigenicity 2; GAL-3, Galectin-3; IL-6, Interleukin-6; TIMP-1, Tissue Inhibitor of Metalloproteinase-1; MMP-9, Matrix Metalloproteinase-9; GDF-15, Growth Differentiation Factor-15; TGF-β1, Transforming Growth Factor-β-1; FGF-23, Fibroblast Growth Factor-23; LAVImax, left atrial maximal volume index; LVEF, left ventricle ejection fraction; LVIDd, left ventricular internal diameter in end-diastole; IVSd, interventricular septal diameter in end-diastole.

Data are presented as median (IQR).

The estimated *P*-values indicate any differences between any groups.

a One missing observation.

b Two missing observations.

c Eight missing observations.

**Table 3.** Baseline Characteristics by Rhythm Outcomes in 164 Participants Undergoing ECV.

	Total	Maintained SR	AF recurrence	*P*-value
Baseline Characteristics	n = 164	n = 74	n = 90	
Male sex	131 (79.9%)	57 (77.0%)	74 (82.2%)	.41
Age, years	66.3 (9.2)	66.4 (9.1)	66.3 (9.4)	.98
mEHRA score				.04
1	10 (6.1%)	1 (1.4%)	9 (10.0%)	
2a	22 (13.4%)	10 (13.5%)	12 (13.3%)	
2b	117 (71.3%)	52 (70.3%)	65 (72.2%)	
3	10 (6.1%)	8 (10.8%)	2 (2.2%)	
4	5 (3.0%)	3 (4.1%)	2 (2.2%)	
Symptom Check List, frequency	15.2 (9.3)	16.7 (9.4)	13.9 (9.1)	.05
Symptom Check List, severity	12.7 (7.9)	14.0 (7.4)	11.7 (8.1)	.07
Hypertension	79 (48.2%)	39 (52.7%)	40 (44.4%)	.29
Ischemic heart disease	17 (10.4%)	10 (13.5%)	7 (7.8%)	.23
Heart failure	47 (28.7%)	25 (33.8%)	22 (24.4%)	.19
Diabetes	11 (6.7%)	3 (4.1%)	8 (8.9%)	.22
TIA	8 (4.9%)	4 (5.4%)	4 (4.4%)	.78
Stroke	14 (8.5%)	6 (8.1%)	8 (8.9%)	.86
Systolic blood pressure (mmHg)	126 (15)	124 (14)	128 (15)	.12
Diastolic blood pressure (mmHg)	81 (12)	81 (12)	82 (12)	.62
Body Mass Index (kg/m^2^)	28.1 (4.6)	28.5 (4.4)	27.7 (4.8)	.32
Ventricular heart rate (BPM)	79 (16)	78 (15)	81 (17)	.36
Reported AF duration before ECV (days)	59 (34-135)	55 (30-107)	63 (41-147)	.07
Antiarrhythmic drugs				
Flecainide	5 (3.0%)	1 (1.4%)	4 (4.4%)	.25
Betablocker	101 (61.6%)	40 (54.1%)	61 (67.8%)	.07
Amiodarone	24 (14.6%)	20 (27.0%)	4 (4.4%)	<.001
Amiodarone^a^	30 (18.3%)	20 (27.0%)	10 (11.1%)	.01
Dronedarone	6 (3.7%)	4 (5.4%)	2 (2.2%)	.28
Non-dihydropyridine calcium channel blocker	3 (1.8%)	0 (0.0%)	3 (3.3%)	.11
Tobacco				.64
Never smoker	70 (42.7%)	29 (39.2%)	41 (45.6%)	
Current smoker	13 (7.9%)	7 (9.5%)	6 (6.7%)	
Previous smoker	81 (49.4%)	38 (51.4%)	43 (47.8%)	
Alcohol units per week	5.0 (2.0-8.0)	5.0 (2.0-7.0)	5.0 (2.0-8.0)	.66

Abbreviations: AF, atrial fibrillation; SR, sinus rhythm; ECV, electrical cardioversion; mEHRA, modified European Heart Rhythm Association symptom classification for AF. 1, None. 2a, Mild, normal activity not affected, not troubled. 2b, Moderate, normal activity not affected, but troubled. 3, Severe, normal activity affected, 4, Disabling, normal activity discontinued. TIA, transient ischemic attack; BPM, beats per minute.

Data are presented as mean (SD) or median (IQR) for continuous measures, and n (%) for categorical measures.

a Including patients with amiodarone startup after ECV.

**Table 4.** Baseline Levels of Circulating and Echocardiographic Biomarkers by Rhythm Outcomes in 164 Participants Undergoing ECV.

	Total	Maintained SR	AF recurrence	*P*-value
Biomarkers	n = 164	n = 74	n = 90	
Routine blood samples				
Hb (g/dL)	15.1 (14.3-16.0)	14.9 (14.1-16.0)	15.3 (14.5-16.0)	.20
CRP (mg/L)	4.0 (3.0-4.0)	4.0 (3.0-4.0)	4.0 (3.0-4.0)	.49
Sodium (mmol/L)	141 (139-142)	141 (139-143)	140 (139-142)	.51
Potassium (mmol/L)^a^	4.2 (3.9-4.4)	4.2 (4.0-4.4)	4.1 (3.9-4.4)	.19
Creatinine (µmol/L)	82 (75-92)	84 (76-92)	82 (75-90)	.41
eGFR (mL/min/1.73m^2^)	81 (70-90)	80 (65-90)	82 (73-90)	.17
Glucose (mmol/L)^a^	5.6 (5.2-6.2)	5.6 (5.2-5.9)	5.7 (5.2-6.3)	.36
HbA1c (mmol/mol)^a^	39 (36-42)	38 (36-42)	39 (36-42)	.52
Cholesterol total (mmol/L)	4.5 (3.8-5.2)	4.3 (3.8-5.2)	4.7 (3.9-5.3)	.45
Triglycerides (mmol/L)	1.1 (0.8-1.6)	1.1 (0.9-1.5)	1.1 (0.8-1.6)	.63
HDL (mmol/L)	1.3 (1.1-1.6)	1.3 (1.1-1.6)	1.3 (1.1-1.6)	.86
LDL (mmol/L)^a^	2.7 (2.1-3.6)	2.7 (2.0-3.6)	2.8 (2.2-3.6)	.58
Hs-TnI (ng/L)^a^	6.8 (4.5-10.1)	6.3 (3.9-9.1)	7.1 (4.9-10.1)	.10
NT-proBNP (ng/L)^a^	1137.8 (691.9-1940.8)	1159.9 (598.0-2020.0)	1137.8 (730.6-1687.1)	.97
Potential novel biomarkers				
PAI-1 activity (IU/mL)^a^	10.4 (3.3-28.9)	11.5 (3.9-29.6)	10.0 (3.2-28.2)	.80
sST-2 (ng/mL)^a^	28.8 (24.0-37.9)	28.8 (23.8-36.6)	30.3 (25.0-38.5)	.48
GAL-3 (ng/mL)^a^	8.39 (6.70-10.64)	8.35 (6.81-10.77)	8.40 (6.53-10.51)	.71
IL-6 (pg/mL)^a^	2.57 (1.66-4.20)	2.57 (1.87-4.05)	2.72 (1.59-4.41)	.99
TIMP-1 (ng/mL)^a^	168 (151-192)	163 (145-182)	172 (153-196)	.03
MMP-9 (ng/mL)^a^	214 (166-293)	199 (153-289)	221 (184-303)	.09
GDF-15 (pg/mL)^a^	1026 (796-1380)	1048 (836-1500)	1003 (776-1356)	.25
TGF-β1 (pg/mL)^b^	2488 (2128-2950)	2408 (1983-2940)	2533 (2256-2954)	.16
FGF-23 (pg/mL)^b^	61 (49-75)	60 (50-77)	62 (49-75)	.71
Echocardiographic biomarkers				
LAVImax, (mL/m^2^)^c^	49 (40-55)	49 (43-57)	48 (39-55)	.16
LVEF (%)^a^	52 (45-54)	52 (45-54)	52 (46-54)	.95
LVIDd (mm)^b^	52 (48-56)	53 (48-57)	52 (47-55)	.11
IVSd (mm)^b^	10 (9-11)	10 (9-11)	9 (9-10)	.13

Abbreviations: Hb, hemoglobin; CRP, C-reactive protein; Estimated GFR, estimated glomerular filtration rate; HbA1c, hemoglobin A1c (glycated hemoglobin); HDL, high-density lipoprotein cholesterol; LDL, low-density lipoprotein cholesterol; hs-TnI, high-sensitivity troponin I; NT-proBNP, N-terminal pro B-type natriuretic peptide; PAI-1 activity, Plasminogen Activator Inhibitor type 1 activity; sST-2, soluble suppression of tumorigenicity 2; GAL-3, Galectin-3; IL-6, Interleukin-6; TIMP-1, Tissue Inhibitor of Metalloproteinase-1; MMP-9, Matrix Metalloproteinase-9; GDF-15, Growth Differentiation Factor-15; TGF-β1, Transforming Growth Factor-β-1; FGF-23, Fibroblast Growth Factor-23; LAVImax, left atrial maximal volume index; LVEF, left ventricle ejection fraction; LVIDd, left ventricular internal diameter in end-diastole; IVSd, interventricular septal diameter in end-diastole.

Data are presented as median (IQR).

The estimated *P*-values indicate any differences between any groups.

a One missing observation.

b Two missing observations.

c Eight missing observations.

### Spontaneous Conversion to SR Versus ECV

Twenty-three of the 24 participants who converted spontaneously to SR were examined while in SR, and blood samples were collected while in SR. The group that spontaneously converted to SR exhibited significantly lower ventricular heart rate, lower prevalence of heart failure, and lower diastolic blood pressure than those who required an ECV (Table 1). They exhibited significantly smaller LAVImax, thinner interventricular septum (IVSd), and higher LVEF than those who underwent ECV. Routine blood samples showed lower creatinine and higher eGFR, higher HDL cholesterol, and lower NT-proBNP levels. Among the potential novel biomarkers, IL-6 and FGF-23 were significantly lower in the participants who spontaneously converted to SR than in those requiring ECV (Table 2).

### Maintained SR Versus AF Recurrence

**Figure 3.**
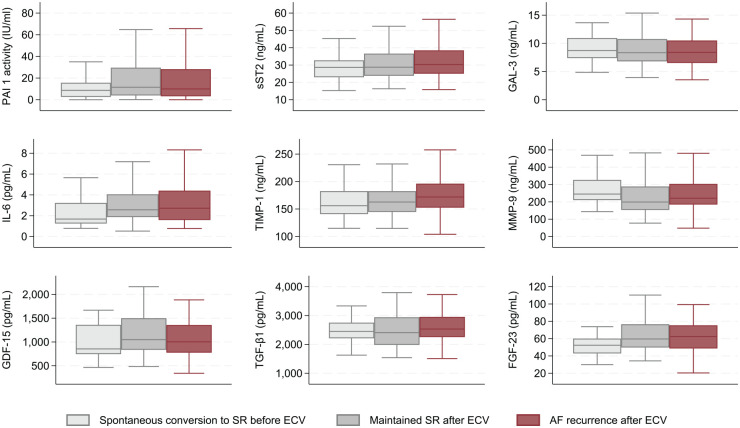
Box plots of baseline biomarker levels in individuals with spontaneous conversion to SR, maintained SR after ECV, and AF recurrence after ECV. Outside values are excluded. Abbreviations: ECV, electrical cardioversion; SR, sinus rhythm; AF, atrial fibrillation; PAI-1 activity, Plasminogen Activator Inhibitor type 1 activity; sST-2, soluble suppression of tumorigenicity 2; GAL-3, Galectin-3; IL-6, Interleukin-6; TIMP-1, Tissue Inhibitor of Metalloproteinase-1; MMP-9, Matrix Metalloproteinase-9; GDF-15, Growth Differentiation Factor-15; TGF-β1, Transforming Growth Factor-β-1; FGF-23, Fibroblast Growth Factor-23.

ECV was performed on 164 participants. Of these, 74 maintained SR throughout the observation period, whereas 90 experienced AF recurrence before leaving the hospital (n = 15) or during the observation period (n = 75). The participants were similar in terms of age, sex, BMI, and comorbidities (Table 3). There was no difference in the measured systolic and diastolic blood pressure between the 2 groups, and the reported AF duration prior to the ECV was similar. The participants who maintained SR after ECV reported more frequent and severe symptoms compared to those with AF recurrence; however, only the symptom frequency was significantly different between the groups. According to mEHRA-scores, more participants with AF recurrence were classified as having no symptoms, while fewer were classified as having severe and disabling symptoms, compared to those maintaining SR. Amiodarone treatment was significantly more prevalent in the participants who maintained SR after ECV than in those with AF recurrence. Routine blood samples were similar between the 2 groups. Among the potential novel biomarkers, TIMP-1 was significantly higher in the AF recurrence group compared to the SR maintenance group, whereas levels of PAI-1 activity, sST2, GAL-3, IL-6, MMP-9, GDF-15, TGF-β1, and FGF-23 did not differ among the groups (Table 4). Box plots of these potential novel biomarkers are presented in Figure 3.

We investigated the relationships between biomarker levels and time to AF recurrence using Kaplan-Meier plots and found no significant associations (Supplemental Figure SF1). Although higher TIMP-1 levels were associated with higher recurrence rates in the Kaplan-Meier plot, the difference was not statistically significant across quartiles or the median.

**Figure 4.**
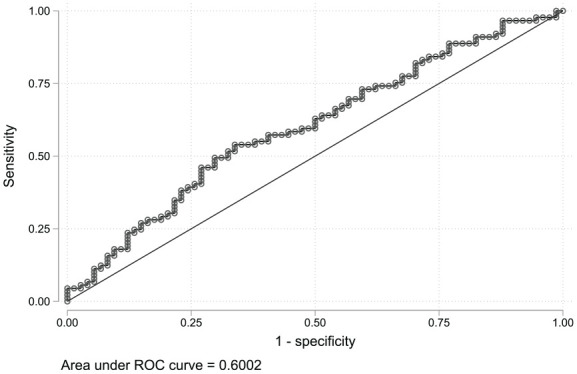
TIMP-1 ROC curve shows the true positive rate (sensitivity) against the false positive rate (1-specificity) at each threshold setting. AUC = 0.6 (CI: 0.5-0.7). Abbreviations: TIMP-1, Tissue Inhibitor of Metalloproteinase-1; ROC, receiver operating characteristics; AUC, area under the curve.

**Figure 5.**
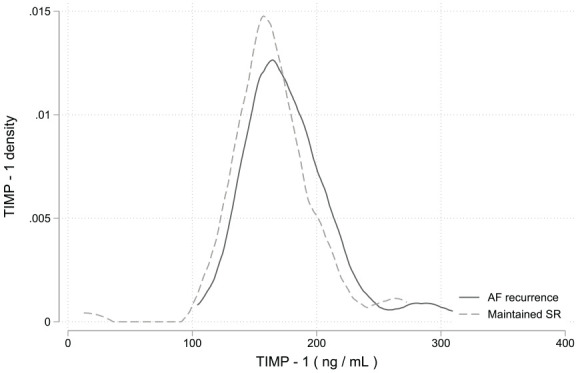
TIMP-1 density plot shows a significant overlap between the AF recurrence group and the group that maintained SR. Abbreviations: TIMP-1, Tissue Inhibitor of Metalloproteinase-1; SR, sinus rhythm; AF, atrial fibrillation.

The area under the curve (AUC) score from the TIMP-1 ROC curve was 0.6 (95% CI: 0.5-0.7; Figure 4). The TIMP-1 density plot showed significant overlap between the AF recurrence group and the SR maintenance group (Figure 5).

The relationships between the circulating biomarkers and baseline clinical characteristics were examined using Spearman’s correlation (rho) analysis (Supplemental Tables S2a and S2b). Several biomarkers correlated significantly with age, and several circulating biomarkers also correlated with each other.

**Figure 6.**
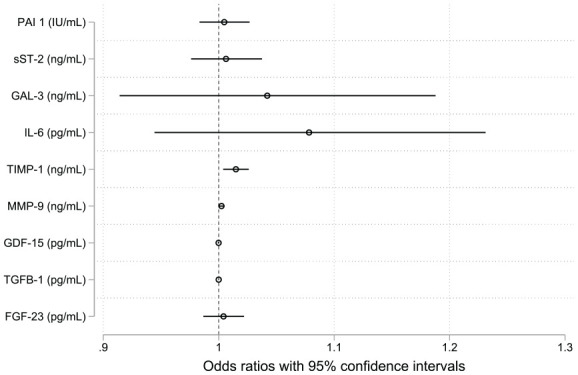
Odds ratios with 95% confidence intervals of the novel biomarkers from multivariate regression models. Each model is adjusted for age, sex, BMI, LAVImax, and NT-proBNP. TIMP-1 was the only biomarker significantly associated with AF recurrence after ECV in the multivariate regression models. Abbreviations: BMI, body mass index; LAVImax, left atrial maximal volume index; NT- proBNP, N-terminal pro-B-type natriuretic peptide; PAI-1 activity, Plasminogen Activator Inhibitor type 1 activity; sST-2, soluble suppression of tumorigenicity 2; GAL-3, Galectin-3; IL-6, Interleukin-6; TIMP-1, Tissue Inhibitor of Metalloproteinase-1; MMP-9, Matrix Metalloproteinase-9; GDF-15, Growth Differentiation Factor-15; TGF-β1, Transforming Growth Factor-β-1; FGF-23, Fibroblast Growth Factor-23.

The potentially novel biomarkers were examined in multivariate logistic regression models adjusted for age, sex, BMI, LAVImax, and NT-proBNP (Figure 6 and Supplemental Tables S3a-i). TIMP-1 remained the only biomarker significantly associated with AF recurrence after ECV in the multivariate logistic regression models. The AUC score from the TIMP-1 ROC curve in the multivariate model showed a slight increase compared to the univariate model, but the difference between the curves was not statistically significant.

## Discussion

In this observational study, we investigated whether a selection of biomarkers obtained before ECV could predict rhythm outcomes in individuals with persistent AF. The main finding of this study is that among the 9 potentially novel biomarkers studied, only TIMP-1 was significantly associated with AF recurrence after ECV.

The participants who spontaneously converted to SR exhibited more favorable cardiovascular profiles than those who required intervention. These findings may indicate better overall health in the former group. Among the potential novel biomarkers, IL-6 and FGF-23 levels were significantly lower in individuals who spontaneously converted to SR compared to those undergoing ECV. Those who successfully maintained SR after ECV reported higher symptom frequency and severity and were more likely to have been treated with amiodarone before the intervention. Among the potential novel biomarkers, TIMP-1 levels were higher in participants with AF recurrence, whereas the other biomarkers showed no significant differences between the 2 groups.

In our study, nearly 55% of the participants who underwent ECV experienced AF recurrence within 4 weeks. This finding is consistent with previous studies^4,5^ and highlights the need for reliable predictors of rhythm outcomes. Inflammation is associated with atrial remodeling, which leads to AF development and progression.^36^ Increased atrial fibrosis is linked to LA conduction abnormalities, which may contribute to the development of AF, and biomarkers associated with fibrosis may be promising predictors of AF.^6-8^

We found that IL-6 levels were significantly lower in those with spontaneous conversion to SR than in those undergoing ECV. Although there was a trend toward higher IL-6 values in the AF recurrence group, this difference was not statistically significant compared to those who maintained SR after ECV. The lower IL-6 levels in participants with spontaneous conversion to SR may suggest less systemic inflammation than in those requiring intervention. In contrast, no detectable difference in IL-6 levels among the participants undergoing ECV may indicate a similar level of systemic inflammation. Several studies have reported higher IL-6 levels as predictive in patients with AF recurrence compared to patients with no recurrence after ECV.^37,38^ However, similar to our findings, other studies have found comparable IL-6 levels between these groups.^39-42^ C-reactive protein (CRP) is an acute inflammatory protein commonly used as a marker of inflammation. Our study found no difference in CRP among any of the groups when measured in routine blood samples. High-sensitivity CRP (hs-CRP) could have provided additional insight but was not measured in our study.

FGF-23 is an early marker of impaired kidney function involved in renal phosphate homeostasis and vitamin D metabolism, and is associated with myocardial fibrosis.^43^ Similar to IL-6, we found that FGF-23 was significantly lower in those with spontaneous conversion to SR compared to those undergoing ECV, with a non-significant trend toward higher FGF-23 values in the individuals with AF recurrence. Lower FGF-23 levels in the participants with spontaneous conversion to SR may indicate better kidney function, which was also observed in our population. FGF-23 levels may also reflect vitamin D status, although we did not measure vitamin D levels in our population. Renal dysfunction is an independent risk factor for AF, and patients with AF are at an increased risk of chronic kidney disease.^44^ A weak association between FGF-23 and AF recurrence has previously been reported,^45^ but FGF-23 levels were not associated with rhythm outcomes in the participants requiring an ECV in our study.

In 23 of the 24 participants who spontaneously converted to SR, biomarkers were collected while in SR. A recent study observed altered biomarker levels in patients with paroxysmal AF compared to those in SR and concluded that it may reflect dynamic pathophysiological changes during acute AF episodes.^46^ Although we only observed the reduced levels of IL-6 and FGF-23 in the group with spontaneous conversion to SR, reversed pathophysiological changes may have resulted in lower biomarker levels in this group.

TIMP-1 alters the extracellular matrix by promoting myocardial fibrosis^47^ and may be associated with increased AF prevalence.^23^ Our study found significantly higher TIMP-1 levels in the participants with AF recurrence compared to those who maintained SR after ECV. Elevated TIMP-1 levels may reflect increased accumulation of fibrotic tissue in individuals with AF recurrence. We found that TIMP-1 levels were lowest in the participants with spontaneous conversion to SR, but this difference was not significant compared to those requiring ECV. The TIMP-1 boxplots showed a significant different central tendency between the participants who maintained SR and those with an AF recurrence. However, overlapping data distribution and AUC score of 0.6 from the TIMP-1 ROC curve suggests a limited clinical utility. Previous studies investigating the association between TIMP-1 and rhythm outcomes found no difference in baseline TIMP-1 levels between those with AF recurrence and those maintaining SR after ECV.^42,48^

None of the other potential novel biomarkers measured in this study differed between the participants with AF recurrence and those who maintained SR. This is to some degree in accordance with the literature (Supplemental Table 1 with references).

Some, but not all, previous studies have shown an association between AF recurrence and LA size.^49,50^ In our study, the participants who spontaneously converted to SR had significantly smaller LAVImax than those who needed an ECV. However, LAVImax was similar between participants with and without AF recurrence.

## Strengths and Limitations

Strengths of the present study include the use of an unselected cohort of patients referred for ECV of persistent AF, which may minimize the risk of selection bias. Additionally, the participants recorded thumb ECGs twice daily, which provided multiple opportunities to detect AF recurrences and reduce the risk of misclassifications.

However, several limitations are present. First, circulating biomarkers were obtained from a single peripheral blood sample, and biomarkers measured in peripheral blood may not be specific to cardiac tissue. Second, the reported AF duration is uncertain as many participants reported an approximate time of AF onset or were diagnosed during routine consultations. Third, the present study is observational and can only describe associations. Fourth, the sample size is relatively small, including only 200 patients. The sample size was selected based on previous studies in the field and the exploratory nature of the current study. It is possible that a larger study could be able to detect additional associations between the tested biomarkers and AF recurrence. However, we considered that the effect of a clinically useful predictor should be detectable in a moderate-sized cohort, and our sample size reflects this rationale. Fifth, the study population was predominantly men of European ancestry, which may limit the generalizability of the study. Lastly, the participants with spontaneous conversion to SR were examined in SR, whereas the other participants were examined in AF. Different heart rhythms may have influenced the results.

## Conclusion

Participants with spontaneous conversion to SR before ECV presented with a more favorable cardiovascular profile than those requiring an ECV. TIMP-1 levels were higher in participants with AF recurrence after ECV than in those who maintained SR, but its predictive ability for AF recurrence was relatively low. PAI-1 activity, sST2, GAL-3, IL-6, MMP-9, GDF-15, TGF-β1, and FGF-23 were not associated with AF recurrence after ECV. Based on these findings, we suggest that these biomarkers should not be used to guide candidate selection for ECV of persistent AF.

## Supplemental Material

sj-docx-1-bmi-10.1177_11772719251361306 – Supplemental material for Circulating Biomarkers and Recurrence of Persistent Atrial Fibrillation After Electrical Cardioversion Supplemental material, sj-docx-1-bmi-10.1177_11772719251361306 for Circulating Biomarkers and Recurrence of Persistent Atrial Fibrillation After Electrical Cardioversion by Elizabeth Lyster Andersen, Magnar Gangås Solberg, Steve Enger, Sophia Onarheim, Mona Olufsen, Trygve Berge, Sissel Åkra, Maiken Kojen Kleveland, Ingrid Elisabeth Christophersen, Sara Reinvik Ulimoen, Ingebjørg Seljeflot and Arnljot Tveit in Biomarker Insights

sj-docx-2-bmi-10.1177_11772719251361306 – Supplemental material for Circulating Biomarkers and Recurrence of Persistent Atrial Fibrillation After Electrical Cardioversion Supplemental material, sj-docx-2-bmi-10.1177_11772719251361306 for Circulating Biomarkers and Recurrence of Persistent Atrial Fibrillation After Electrical Cardioversion by Elizabeth Lyster Andersen, Magnar Gangås Solberg, Steve Enger, Sophia Onarheim, Mona Olufsen, Trygve Berge, Sissel Åkra, Maiken Kojen Kleveland, Ingrid Elisabeth Christophersen, Sara Reinvik Ulimoen, Ingebjørg Seljeflot and Arnljot Tveit in Biomarker Insights
