# Supplementary material for: Circulating Biomarkers and Recurrence of Persistent Atrial Fibrillation After Electrical Cardioversion
Source: Biomark Insights. 2025 Sep 11;20:11772719251361306. doi: 10.1177/11772719251361306 (PMC12432329; doi:10.1177/11772719251361306)
Supplement: sj-docx-1-bmi-10.1177_11772719251361306 – Supplemental material for Circulating Biomarkers and Recurrence of Persistent Atrial Fibrillation After Electrical Cardioversion [file sj-docx-1-bmi-10.1177_11772719251361306.docx]

##### **Supplementary Table S1.** Circulating biomarkers and prediction of AF recurrence after electrical cardioversion.

| **Biomarker** | **Author** | **Year** | **Findings** |
| --- | --- | --- | --- |
| PAI-1 activity | Tveit et al.^1^ | 2008 | High PAI-1 activity levels at baseline predicted AF recurrence after electrical cardioversion. |
|  | Andersson et al.^2^ | 2011 | PAI-1 activity levels at baseline were not associated with rhythm outcomes after electrical cardioversion. |
| sST2 | Walek et al.^3^ | 2020 | Lower levels of sST2 predicted SR maintenance at 12 months after electrical cardioversion. |
|  | Merino-Merino et al.^4^ | 2021 | No significant difference in baseline ST2 levels in patients with AF recurrence and SR maintenance after electrical cardioversion. |
|  | García-Seara et al.^5^ | 2023 | Baseline ST-2 did not predict AF recurrence after electrical cardioversion or PVI. |
| GAL-3 | Begg et al.^6^ | 2017 | Gal-3 levels did not predict AF recurrence after electrical cardioversion. |
|  | Gürses et al.^7^ | 2019 | Gal-3 levels were higher in patients with AF recurrence. |
|  | Walek et al.^3^ | 2020 | Gal-3 levels did not differ between SR maintenance and AF recurrence group after electrical cardioversion. |
|  | Cichon M. et al.^8^ | 2021 | GAL-3 levels were not associated with rhythm outcomes four weeks after electrical cardioversion. |
|  | Pauklin et al.^9^ | 2022 | Gal-3 levels did not differ between SR maintenance and AF recurrence group. |
| IL-6 | Tveit et al.^10^ | 2007 | Baseline IL-6 levels did not differ between SR maintenance and AF recurrence group. |
|  | Henningsen et al.^11^ | 2009 | IL-6 predicted AF recurrence rate after electrical cardioversion. |
|  | Leftheriotis et al.^12^ | 2009 | No significant difference in IL-6 levels between recurrence and no recurrence group. |
|  | Kawamura et al.^13^ | 2012 | Il-6 at baseline did not differ between SR maintenance and AF recurrence group. |
|  | Smit et al.^14^ | 2012 | Elevated IL-6 levels were associated with AF recurrence after electrical cardioversion. |
|  | Lewicka et al.^15^ | 2016 | IL-6 levels at baseline did not differ between SR maintenance and AF recurrence group. |
|  | Mlodawska et al.^16^ | 2016 | IL-6 at baseline did not differ between SR maintenance and AF recurrence group. |
| TIMP-1 | Climent et al.^17^ | 2009 | No significant difference in baseline TIMP-1 between patients who maintained SR and patients with AF recurrence after electrical cardioversion. |
|  | Mukherjee et al.^18^ | 2013 | TIMP-1 at baseline did not differ between SR maintenance and AF recurrence group. |
|  | Mlodawska et al.^16^ | 2016 | TIMP-1 at baseline did not differ between SR maintenance and AF recurrence group. |
| MMP-9 | Climent et al.^17^ | 2009 | No significant difference in baseline MMP-9 between patients who maintained SR and patients with AF recurrence after electrical cardioversion. |
|  | Kim et al.^19^ | 2009 | Baseline MMP-9 levels did not differ between the groups with and without AF recurrence after successful cardioversion. |
|  | Mukherjee et al.^18^ | 2013 | Baseline MMP-9 was significantly higher in patients with AF recurrence after electrical cardioversion. MMP-9 was a predictor for AF recurrence. |
| GDF-15 | Cichon et al.^8^ | 2021 | GDF-15 levels were not associated with electrical cardioversion efficacy. |
| TGF-β1 | Kim et al.^19^ | 2009 | The TGF-β levels did not differ between the groups with and without AF recurrence after successful cardioversion. |
| FGF-23 | Begg et al.^6^ | 2017 | FGF-23 was weakly associated with AF recurrence after electrical cardioversion. |
| Hs-TnI | Horjen et al.^20^ | 2016 | Baseline hs-TnI levels did not differ between the SR maintenance and the AF recurrence group after electrical cardioversion. |
| NT-proBNP | Tveit et al.^21^ | 2009 | Baseline NT-proBNP levels were not associated with rhythm outcomes after electrical cardioversion. |
|  | Kallergis et al. ^22^ | 2010 | Baseline NT-proBNP levels were significantly higher in patients with AF recurrence after electrical cardioversion. |
|  | Mukherjee et al.^18^ | 2013 | Baseline NT-proBNP levels was significantly lower in the SR maintenance group compare to the AF recurrence group. |
|  | Pauklin et al.^9^ | 2022 | Baseline NT-proBNP levels was significantly lower in the SR maintenance group compare to the AF recurrence group. |

##### Abbreviations: PAI-1 activity, Plasminogen Activator Inhibitor type 1 activity; sST2, soluble suppression of tumorigenicity 2; GAL-3, Galectin-3; IL-6, Interleukin-6; TIMP-1, Tissue Inhibitor of Metalloproteinase-1; MMP-9, Matrix Metalloproteinase-9; GDF-15, Growth Differentiation Factor-15; TGF-β1, Transforming Growth Factor-β-1; FGF-23, Fibroblast Growth Factor-23; Hs-TnI, high sensitive troponin I; NT-proBNP, N-terminal pro B-type natriuretic peptide.

##### **Supplementary Table S2a.** Spearman’s correlation coefficient rho to baseline biomarkers and characteristics with corresponding Bonferroni adjusted p-values at 0.05 significance level.

|  | PAI-1^†^ | *P* value | sST-2^†^ | *P* value | GAL-3^†^ | *P* value | IL-6^†^ | *P* value | TIMP-1^†^ | *P* value | MMP-9^†^ | *P* value | GDF-15^†^ | *P* value | TGF-β1 ^‡^ | *P* value | FGF-23^‡^ | *P* value |
| --- | --- | --- | --- | --- | --- | --- | --- | --- | --- | --- | --- | --- | --- | --- | --- | --- | --- | --- |
| Age, years | **-0.24*** | **0.030** | -0.04 | 1.000 | **0.27*** | **0.007** | **0.36*** | **<0.001** | 0.21 | 0.104 | -0.06 | 1.000 | **0.56*** | **<0.001** | 0.06 | 1.000 | 0.08 | 1.000 |
| Systolic blood pressure, mmHg | 0.11 | 1.000 | -0.01 | 1.000 | 0.11 | 1.000 | 0.15 | 1.000 | 0.16 | 1.000 | 0.03 | 1.000 | 0.11 | 1.000 | -0.06 | 1.000 | 0.12 | 1.000 |
| Diastolic blood pressure, mmHg | 0.15 | 1.000 | 0.02 | 1.000 | 0.02 | 1.000 | 0.11 | 1.000 | 0.04 | 1.000 | 0.02 | 1.000 | <-0.01 | 1.000 | -0.01 | 1.000 | 0.11 | 1.000 |
| Symptom Check List, frequency | -0.05 | 1.000 | -0.03 | 1.000 | 0.22 | 0.0661 | 0.13 | 1.000 | 0.07 | 1.000 | 0.04 | 1.000 | 0.15 | 1.000 | -0.03 | 1.000 | -0.02 | 1.000 |
| Symptom Check List, severity | -0.04 | 1.000 | -0.02 | 1.000 | 0.20 | 0.1889 | 0.11 | 1.000 | 0.05 | 1.000 | 0.02 | 1.000 | 0.14 | 1.000 | -0.03 | 1.000 | -0.01 | 1.000 |
| mEHRA score | 0.002 | 1.000 | 0.07 | 1.000 | 0.10 | 1.000 | -0.04 | 1.000 | 0.01 | 1.000 | 0.04 | 1.000 | 0.05 | 1.000 | -0.07 | 1.000 | -0.02 | 1.000 |
| Previous ECVs | 0.09 | 1.000 | -0.04 | 1.000 | -0.12 | 1.000 | -0.08 | 1.000 | -0.14 | 1.000 | -0.01 | 1.000 | -0.07 | 1.000 | -0.10 | 1.000 | 0.05 | 1.000 |
| Body mass index, kg/m2 | **0.49*** | **<0.001** | 0.03 | 1.000 | 0.06 | 1.000 | **0.23*** | **0.039** | 0.02 | 1.000 | 0.12 | 1.000 | -0.03 | 1.000 | 0.10 | 1.000 | 0.10 | 1.000 |
| Heart rate | 0.11 | 1.000 | 0.05 | 1.000 | 0.02 | 1.000 | **0.31*** | **<0.001** | 0.17 | 0.842 | **0.25*** | **0.018** | 0.18 | 0.562 | -0.01 | 1.000 | 0.07 | 1.000 |
| Sex | 0.05 | 1.000 | **0.25*** | **0.016** | **-0.31*** | **<0.001** | -0.04 | 1.000 | -0.17 | 0.491 | -0.02 | 1.000 | -0.09 | 1.000 | -0.12 | 1.000 | -0.09 | 1.000 |
| Hypertension | 0.13 | 1.000 | 0.11 | 1.000 | 0.01 | 1.000 | 0.12 | 1.000 | 0.08 | 1.000 | -<0.01 | 1.000 | 0.20 | 1.000 | -0.01 | 1.000 | 0.22 | 0.055 |
| Cardiovascular disease | 0.08 | 1.000 | 0.08 | 1.000 | 0.06 | 1.000 | 0.01 | 1.000 | 0.07 | 1.000 | -0.03 | 1.000 | 0.17 | 1.000 | 0.08 | 1.000 | <-0.01 | 1.000 |
| Congestive Heart Failure | 0.05 | 1.000 | **0.24*** | **0.025** | 0.06 | 1.000 | 0.20 | 0.156 | -0.03 | 1.000 | 0.11 | 1.000 | **0.26*** | **0.006** | 0.06 | 1.000 | 0.14 | 1.000 |
| Diabetes Mellitus | -0.09 | 1.000 | <0.01 | 1.000 | 0.06 | 1.000 | 0.05 | 1.000 | 0.11 | 1.000 | 0.11 | 1.000 | **0.29*** | **0.001** | <-0.01 | 1.000 | 0.09 | 1.000 |
| Transient Ischemic Attack | -0.01 | 1.000 | 0.12 | 1.000 | -0.10 | 1.000 | 0.02 | 1.000 | -0.13 | 1.000 | -0.13 | 1.000 | <-0.01 | 1.000 | -0.04 | 1.000 | -0.09 | 1.000 |
| Stroke | -0.15 | 1.000 | 0.04 | 1.000 | 0.04 | 1.000 | 0.06 | 1.000 | -0.03 | 1.000 | -0.02 | 1.000 | 0.07 | 1.000 | 0.08 | 1.000 | 0.03 | 1.000 |
| Chronic Obstructive Pulmonary disease/Emphysema | 0.07 | 1.000 | -0.14 | 1.000 | 0.14 | 1.000 | 0.05 | 1.000 | 0.09 | 1.000 | 0.13 | 1.000 | 0.14 | 1.000 | 0.01 | 1.000 | -0.06 | 1.000 |

^†^ One missing observation ^‡^ Two missing observations.

##### Abbreviations: PAI-1 activity, Plasminogen Activator Inhibitor type 1 activity; sST-2, soluble suppression of tumorigenicity 2; GAL-3, Galectin-3; IL-6, Interleukin-6; TIMP-1, Tissue Inhibitor of Metalloproteinase-1; MMP-9, Matrix Metalloproteinase-9; GDF-15, Growth Differentiation Factor-15; TGF-β1, Transforming Growth Factor-β-1; FGF-23, Fibroblast Growth Factor-23; mEHRA score, modified European Heart Rhythm Association symptom classification for AF ; ECV, Electrical Cardioversion.

##### **Supplementary Table S2b.** Spearman’s correlation coefficient rho with corresponding Bonferroni adjusted p-values at 0.05 significance level.

| PAI-1^†^ | 1.00 |  |  |  |  |  |  |  |  |  |  |
| --- | --- | --- | --- | --- | --- | --- | --- | --- | --- | --- | --- |
|  |  |  |  |  |  |  |  |  |  |  |  |
| sST-2^†^ | -0.10 | 1.00 |  |  |  |  |  |  |  |  |  |
|  | 1.000 |  |  |  |  |  |  |  |  |  |  |
| GAL-3^†^ | -0.13 | -0.02 | 1.00 |  |  |  |  |  |  |  |  |
|  | 1.000 | 1.000 |  |  |  |  |  |  |  |  |  |
| IL-6^†^ | 0.11 | 0.21 | **0.28*** | 1.00 |  |  |  |  |  |  |  |
|  | 1.000 | 0.107 | **0.002** |  |  |  |  |  |  |  |  |
| TIMP-1^†^ | 0.09 | 0.05 | 0.22 | **0.43*** | 1.00 |  |  |  |  |  |  |
|  | 1.000 | 1.000 | 0.051 | **<0.001** |  |  |  |  |  |  |  |
| MMP-9^†^ | 0.17 | -0.03 | 0.06 | **0.24*** | **0.25*** | 1.00 |  |  |  |  |  |
|  | 0.549 | 1.000 | 1.000 | **0.024** | **0.012** |  |  |  |  |  |  |
| GDF-15^†^ | -0.13 | 0.18 | **0.34*** | **0.50*** | **0.30*** | <-0.01 | 1.00 |  |  |  |  |
|  | 1.000 | 0.421 | **0.000** | **<0.001** | **<0.001** | 1.000 |  |  |  |  |  |
| TGF-β1^‡^ | 0.06 | 0.04 | 0.09 | 0.22 | 0.22 | 0.08 | 0.11 | 1.00 |  |  |  |
|  | 1.000 | 1.000 | 1.000 | 0.068 | 0.076 | 1.000 | 1.000 |  |  |  |  |
| FGF-23^‡^ | 0.07 | 0.06 | 0.10 | 0.21 | 0.17 | 0.02 | 0.18 | 0.07 | 1.00 |  |  |
|  | 1.000 | 1.000 | 1.000 | 0.085 | 0.647 | 1.000 | 0.388 | 1.000 |  |  |  |
| Hs-TnI | -0.09  1.000 | **0.2688***  **0.007** | 0.01  1.000 | 0.22  0.082 | 0.22  0.090 | 0.04  1.000 | **0.37***  **0.000** | 0.18  0.588 | 0.15  1.000 | 1.00 |  |
| NT-  proBNP | -0.13  1.000 | 0.23  0.057 | 0.21  0.207 | **0.41***  **<0.001** | **0.27***  **0.005** | -0.06  1.000 | **0.46***  **<0.001** | 0.16  1.000 | **0.24***  **0.045** | **0.29***  **0.002** | 1.00 |
|  | PAI-1^†^ | sST-2^†^ | GAL-3^†^ | IL-6^†^ | TIMP-1^†^ | MMP-9^†^ | GDF-15^†^ | TGF-β1^‡^ | FGF-23^‡^ | Hs-TnI | NT-  proBNP |

^†^ One missing observation ^‡^ Two missing observations.

##### Abbreviations: PAI-1 activity, Plasminogen Activator Inhibitor type 1 activity; sST-2, soluble suppression of tumorigenicity 2; GAL-3, Galectin-3; IL-6, Interleukin-6; TIMP-1, Tissue Inhibitor of Metalloproteinase-1; MMP-9, Matrix Metalloproteinase-9; GDF-15, Growth Differentiation Factor-15; TGF-β1, Transforming Growth Factor-β-1; FGF-23, Fibroblast Growth Factor-23; Hs-TnI, High sensitive troponin I; NT pro-BNP, N terminal pro b-type natriuretic peptide.

##### **Supplementary Tables S3a-i.** Odds ratios with 95% confidence intervals from multivariate logistic regression models of new biomarkers adjusted for age, sex, BMI, LAVImax and NT-proBNP.

##### Table S3a.

|  | Odds ratio | Std. err. | z | P>z | [95% conf. | interval] |
| --- | --- | --- | --- | --- | --- | --- |
| PAI-1 activity | 1.004796 | 0.0110606 | 0.43 | 0.664 | 0.9833495 | 1.02671 |
| Age | 0.9989347 | 0.0191736 | -0.06 | 0.956 | 0.9620533 | 1.03723 |
| Male sex | 1.305126 | 0.603197 | 0.58 | 0.564 | 0.5275314 | 3.228916 |
| BMI | 0.9474219 | 0.0432964 | -1.18 | 0.237 | 0.8662518 | 1.036198 |
| LAVImax | 0.9792727 | 0.0141732 | -1.45 | 0.148 | 0.9518841 | 1.007449 |
| NT-proBNP | 1.000017 | 0.0001621 | 0.1 | 0.918 | 0.999699 | 1.000335 |
| _cons | 12.69444 | 24.22863 | 1.33 | 0.183 | 0.3013069 | 534.8331 |

##### Table S3b.

|  | Odds ratio | Std. err. | z | P>z | [95% conf. | interval] |
| --- | --- | --- | --- | --- | --- | --- |
| sST-2 | 1.006336 | 0.0156663 | 0.41 | 0.685 | 0.9760941 | 1.037515 |
| Age | 0.9991618 | 0.019291 | -0.04 | 0.965 | 0.9620587 | 1.037696 |
| Male sex | 1.229161 | 0.5775146 | 0.44 | 0.661 | 0.4894143 | 3.087032 |
| BMI | 0.9576004 | 0.0378222 | -1.1 | 0.273 | 0.886267 | 1.034675 |
| LAVImax | 0.9789199 | 0.0142078 | -1.47 | 0.142 | 0.9514654 | 1.007167 |
| NT-proBNP | 0.9999837 | 0.000163 | -0.1 | 0.92 | 0.9996644 | 1.000303 |
| _cons | 9.229152 | 18.43758 | 1.11 | 0.266 | 0.1839373 | 463.0776 |

##### Table S3c.

|  | Odds ratio | Std. err. | z | P>z | [95% conf. | interval] |
| --- | --- | --- | --- | --- | --- | --- |
| GAL-3 | 1.042051 | 0.0696201 | 0.62 | 0.538 | 0.9141544 | 1.187841 |
| Age | 0.9954251 | 0.0193589 | -0.24 | 0.814 | 0.9581963 | 1.0341 |
| Male sex | 1.361972 | 0.6426675 | 0.65 | 0.513 | 0.5401518 | 3.434162 |
| BMI | 0.9540214 | 0.0379026 | -1.18 | 0.236 | 0.8825524 | 1.031278 |
| LAVImax | 0.9791028 | 0.0142322 | -1.45 | 0.146 | 0.9516017 | 1.007399 |
| NT-proBNP | 0.999983 | 0.0001603 | -0.11 | 0.915 | 0.9996688 | 1.000297 |
| _cons | 10.2753 | 19.68843 | 1.22 | 0.224 | 0.2403325 | 439.315 |

##### Table S3d.

|  | Odds ratio | Std. err. | z | P>z | [95% conf. | interval] |
| --- | --- | --- | --- | --- | --- | --- |
| IL-6 | 1.078288 | 0.0729516 | 1.11 | 0.265 | 0.9443803 | 1.231184 |
| Age | 0.9932614 | 0.019239 | -0.35 | 0.727 | 0.9562605 | 1.031694 |
| Male sex | 1.195149 | 0.5576179 | 0.38 | 0.702 | 0.4789375 | 2.982395 |
| BMI | 0.9480884 | 0.0388535 | -1.3 | 0.193 | 0.8749149 | 1.027382 |
| LAVImax | 0.9822931 | 0.0144576 | -1.21 | 0.225 | 0.9543615 | 1.011042 |
| NT-proBNP | 0.9998908 | 0.0001777 | -0.61 | 0.539 | 0.9995425 | 1.000239 |
| _cons | 16.80637 | 32.441 | 1.46 | 0.144 | 0.3823108 | 738.8072 |

##### Table S3e.

|  | Odds ratio | Std. err. | z | P>z | [95% conf. | interval] |
| --- | --- | --- | --- | --- | --- | --- |
| TIMP-1 | 1.014897 | 0.0056502 | 2.66 | 0.008 | 1.003883 | 1.026032 |
| Age | 0.989117 | 0.0198024 | -0.55 | 0.585 | 0.9510565 | 1.028701 |
| Male sex | 1.30927 | 0.6213185 | 0.57 | 0.57 | 0.5165216 | 3.318713 |
| BMI | 0.9493896 | 0.0388353 | -1.27 | 0.204 | 0.8762451 | 1.02864 |
| LAVImax | 0.9847817 | 0.0148134 | -1.02 | 0.308 | 0.9561718 | 1.014248 |
| NT-proBNP | 0.9998831 | 0.0001667 | -0.7 | 0.483 | 0.9995565 | 1.00021 |
| _cons | 1.869654 | 3.862361 | 0.3 | 0.762 | 0.0326089 | 107.198 |

##### Table S3f.

|  | Odds ratio | Std. err. | z | P>z | [95% conf. | interval] |
| --- | --- | --- | --- | --- | --- | --- |
| MMP-9 | 1.002439 | 0.0013696 | 1.78 | 0.075 | 0.9997583 | 1.005127 |
| Age_ | 1.001536 | 0.0192916 | 0.08 | 0.936 | 0.9644301 | 1.04007 |
| Male | 1.320525 | 0.6150244 | 0.6 | 0.551 | 0.5300367 | 3.289934 |
| BMI2 | 0.9467218 | 0.0382765 | -1.35 | 0.176 | 0.8745968 | 1.024795 |
| LAESV_V1 | 0.9817476 | 0.014374 | -1.26 | 0.208 | 0.9539754 | 1.010328 |
| nBNP_V1 | 0.999968 | 0.0001618 | -0.2 | 0.843 | 0.999651 | 1.000285 |
| _cons | 6.122906 | 11.86688 | 0.93 | 0.35 | 0.1371618 | 273.3266 |

##### Table S3g.

|  | Odds ratio | Std. err. | z | P>z | [95% conf. | interval] |
| --- | --- | --- | --- | --- | --- | --- |
| GDF-15 | 0.9998834 | 0.0003514 | -0.33 | 0.74 | 0.999195 | 1.000572 |
| Age_ | 1.000664 | 0.0208901 | 0.03 | 0.975 | 0.9605464 | 1.042457 |
| Male | 1.319559 | 0.619954 | 0.59 | 0.555 | 0.5254343 | 3.313901 |
| BMI2 | 0.9569962 | 0.0377247 | -1.12 | 0.265 | 0.8858413 | 1.033867 |
| LAESV_V1 | 0.9793279 | 0.0142306 | -1.44 | 0.151 | 0.9518298 | 1.00762 |
| nBNP_V1 | 1.000026 | 0.0001765 | 0.15 | 0.882 | 0.9996803 | 1.000372 |
| _cons | 10.37963 | 20.17357 | 1.2 | 0.229 | 0.2300435 | 468.3322 |

##### Table S3h.

|  | Odds ratio | Std. err. | z | P>z | [95% conf. | interval] |
| --- | --- | --- | --- | --- | --- | --- |
| TGF-β1 | 0.9999811 | 0.0001016 | -0.19 | 0.852 | 0.9997819 | 1.00018 |
| Age | 0.9965347 | 0.0190332 | -0.18 | 0.856 | 0.95992 | 1.034546 |
| Male sex | 1.320467 | 0.6129112 | 0.6 | 0.549 | 0.5316575 | 3.279619 |
| BMI | 0.957191 | 0.0378795 | -1.11 | 0.269 | 0.8857548 | 1.034389 |
| LAVImax | 0.9795353 | 0.0143441 | -1.41 | 0.158 | 0.9518209 | 1.008057 |
| NT-proBNP | 0.9999851 | 0.0001603 | -0.09 | 0.926 | 0.9996708 | 1.000299 |
| _cons | 12.94076 | 24.93931 | 1.33 | 0.184 | 0.2961638 | 565.4411 |

##### Table S3i.

|  | Odds ratio | Std. err. | z | P>z | [95% conf. | interval] |
| --- | --- | --- | --- | --- | --- | --- |
| FGF-23 | 1.004165 | 0.0089968 | 0.46 | 0.643 | 0.9866857 | 1.021954 |
| Age | 0.9956471 | 0.0190853 | -0.23 | 0.82 | 0.9589346 | 1.033765 |
| Male sex | 1.370758 | 0.6380376 | 0.68 | 0.498 | 0.5505002 | 3.413217 |
| BMI | 0.9551063 | 0.0377907 | -1.16 | 0.246 | 0.8838369 | 1.032122 |
| LAVImax | 0.979424 | 0.0142522 | -1.43 | 0.153 | 0.9518849 | 1.00776 |
| NT-proBNP | 0.9999802 | 0.0001591 | -0.12 | 0.901 | 0.9996684 | 1.000292 |
| _cons | 10.47548 | 20.21228 | 1.22 | 0.223 | 0.2386679 | 459.7843 |

##### Abbreviations: CIs, confidence intervals; BMI, body mass index; LAVImax, left atrial maximal volume index; NT- proBNP, N-terminal pro-B-type natriuretic peptide; PAI-1 activity, Plasminogen Activator Inhibitor type 1 activity; sST-2, soluble suppression of tumorigenicity 2; GAL-3, Galectin-3; IL-6, Interleukin-6; TIMP-1, Tissue Inhibitor of Metalloproteinase-1; MMP-9, Matrix Metalloproteinase-9; GDF-15, Growth Differentiation Factor-15; TGF-β1, Transforming Growth Factor-β-1; FGF-23, Fibroblast Growth Factor-23.

##### **Supplementary Figure SF1.** Kaplan-Meier plots of days to AF recurrence by TIMP-1 quartiles and median.


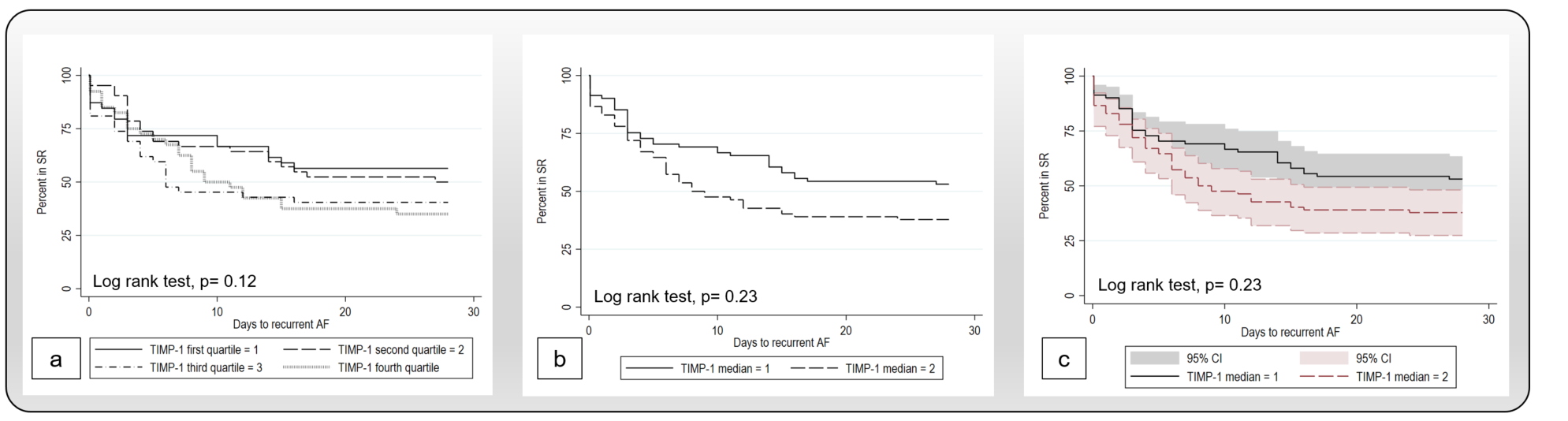


##### Kaplan-Meier plots of days to AF recurrence by TIMP-1 quartiles (a), by median (b), and by median with confidence interval (c).

##### Abbreviations: SR, sinus rhythm; AF, atrial fibrillation; TIMP-1, Tissue Inhibitor of Metalloproteinase-1; CI, confidence interval.

1. Tveit A, Seljeflot I, Grundvold I, Abdelnoor M, Smith P, Arnesen H. Levels of PAI-1 and outcome after electrical cardioversion for atrial fibrillation. Thrombosis research. 2008;121(4):447-53.

2. Andersson J, Almroth H, Höglund N, Jensen S, Tornvall P, Englund A, et al. Markers of fibrinolysis as predictors for maintenance of sinus rhythm after electrical cardioversion. Thrombosis research. 2011;127(3):189-92.

3. Wałek P, Gorczyca I, Grabowska U, Spałek M, Wożakowska-Kapłon B. The prognostic value of soluble suppression of tumourigenicity 2 and galectin-3 for sinus rhythm maintenance after cardioversion due to persistent atrial fibrillation in patients with normal left ventricular systolic function. Europace : European pacing, arrhythmias, and cardiac electrophysiology : journal of the working groups on cardiac pacing, arrhythmias, and cardiac cellular electrophysiology of the European Society of Cardiology. 2020;22(10):1470-9.

4. Merino-Merino A, Saez-Maleta R, Salgado-Aranda R, AlKassam-Martinez D, Pascual-Tejerina V, Martin-González J, et al. When should we measure biomarkers in patients with atrial fibrillation to predict recurrences? Am J Emerg Med. 2021;39:248-9.

5. García-Seara J, González Melchor L, Rodríguez García J, Gude F, Martínez Sande JL, Rodríguez Mañero M, et al. Role of Soluble ST2 Biomarker in Predicting Recurrence of Atrial Fibrillation after Electrical Cardioversion or Pulmonary Vein Isolation. Int J Mol Sci. 2023;24(18).

6. Begg GA, Lip GY, Plein S, Tayebjee MH. Circulating biomarkers of fibrosis and cardioversion of atrial fibrillation: A prospective, controlled cohort study. Clin Biochem. 2017;50(1-2):11-5.

7. Gürses KM, Yalçın MU, Koçyiğit D, Canpınar H, Ateş AH, Canpolat U, et al. Serum galectin-3 level predicts early recurrence following successful direct-current cardioversion in persistent atrial fibrillation patients. Turk Kardiyol Dern Ars. 2019;47(7):564-71.

8. Cichoń M, Mizia-Szubryt M, Olszanecka-Glinianowicz M, Bożentowicz-Wikarek M, Owczarek AJ, Michalik R, Mizia-Stec K. Biomarkers of left atrial overload in obese and nonobese patients with atrial fibrillation qualified for electrical cardioversion. Kardiol Pol. 2021;79(3):269-76.

9. Pauklin P, Zilmer M, Eha J, Tootsi K, Kals M, Kampus P. Markers of Inflammation, Oxidative Stress, and Fibrosis in Patients with Atrial Fibrillation. Oxid Med Cell Longev. 2022;2022:4556671.

10. Tveit A, Seljeflot I, Grundvold I, Abdelnoor M, Smith P, Arnesen H. Effect of candesartan and various inflammatory markers on maintenance of sinus rhythm after electrical cardioversion for atrial fibrillation. Am J Cardiol. 2007;99(11):1544-8.

11. Henningsen KM, Therkelsen SK, Bruunsgaard H, Krabbe KS, Pedersen BK, Svendsen JH. Prognostic impact of hs-CRP and IL-6 in patients with persistent atrial fibrillation treated with electrical cardioversion. Scand J Clin Lab Invest. 2009;69(3):425-32.

12. Leftheriotis DI, Fountoulaki KT, Flevari PG, Parissis JT, Panou FK, Andreadou IT, et al. The predictive value of inflammatory and oxidative markers following the successful cardioversion of persistent lone atrial fibrillation. International journal of cardiology. 2009;135(3):361-9.

13. Kawamura M, Munetsugu Y, Kawasaki S, Onishi K, Onuma Y, Kikuchi M, et al. Type III procollagen-N-peptide as a predictor of persistent atrial fibrillation recurrence after cardioversion. Europace : European pacing, arrhythmias, and cardiac electrophysiology : journal of the working groups on cardiac pacing, arrhythmias, and cardiac cellular electrophysiology of the European Society of Cardiology. 2012;14(12):1719-25.

14. Smit MD, Maass AH, De Jong AM, Muller Kobold AC, Van Veldhuisen DJ, Van Gelder IC. Role of inflammation in early atrial fibrillation recurrence. Europace : European pacing, arrhythmias, and cardiac electrophysiology : journal of the working groups on cardiac pacing, arrhythmias, and cardiac cellular electrophysiology of the European Society of Cardiology. 2012;14(6):810-7.

15. Lewicka E, Dudzinska-Gehrmann J, Dabrowska-Kugacka A, Zagozdzon P, Lizewska A, Danilowicz-Szymanowicz L, Raczak G. Neopterin and interleukin-6 as predictors of recurrent atrial fibrillation. Anatol J Cardiol. 2016;16(8):563-71.

16. Mlodawska E, Tomaszuk-Kazberuk A, Lopatowska P, Waszkiewicz E, Bachorzewska-Gajewska H, Malyszko J, et al. Matrix Metalloproteinase Neutrophil Gelatinase-Associated Lipocalin Complex Predicts Atrial Fibrillation Recurrence after Electrical Cardioversion in Obese Patients. Cardiorenal Med. 2016;7(1):11-20.

17. Climent V, Marín F, Mainar L, Roldán V, García A, Martínez JG, Lip GY. Influence of electrical cardioversion on inflammation and indexes of structural remodeling, in persistent atrial fibrillation. International journal of cardiology. 2009;132(2):227-32.

18. Mukherjee R, Akar JG, Wharton JM, Adams DK, McClure CD, Stroud RE, et al. Plasma profiles of matrix metalloproteinases and tissue inhibitors of the metalloproteinases predict recurrence of atrial fibrillation following cardioversion. J Cardiovasc Transl Res. 2013;6(4):528-35.

19. Kim SK, Pak HN, Park JH, Ko KJ, Lee JS, Choi JI, et al. Clinical and serological predictors for the recurrence of atrial fibrillation after electrical cardioversion. Europace : European pacing, arrhythmias, and cardiac electrophysiology : journal of the working groups on cardiac pacing, arrhythmias, and cardiac cellular electrophysiology of the European Society of Cardiology. 2009;11(12):1632-8.

20. Horjen AW, Ulimoen SR, Seljeflot I, Smith P, Arnesen H, Norseth J, Tveit A. High-Sensitivity Troponin I and Rhythm Outcome after Electrical Cardioversion for Persistent Atrial Fibrillation. Cardiology. 2016;133(4):233-8.

21. Tveit A, Seljeflot I, Grundvold I, Abdelnoor M, Arnesen H, Smith P. Candesartan, NT-proBNP and recurrence of atrial fibrillation after electrical cardioversion. International journal of cardiology. 2009;131(2):234-9.

22. Kallergis EM, Manios EG, Kanoupakis EM, Mavrakis HE, Goudis CA, Maliaraki NE, et al. Effect of sinus rhythm restoration after electrical cardioversion on apelin and brain natriuretic Peptide prohormone levels in patients with persistent atrial fibrillation. Am J Cardiol. 2010;105(1):90-4.
